# Supplementary material for: Radiomics Based on Nomogram Predict Pelvic Lymphnode Metastasis in Early-Stage Cervical Cancer
Source: Diagnostics (Basel). 2022 Oct 10;12(10):2446. doi: 10.3390/diagnostics12102446 (PMC9600299; doi:10.3390/diagnostics12102446)
Supplement: Supplementary file 1 [file diagnostics-12-02446-s001.zip › Supplementary S1. The formula of the radiomics signature .pdf]

Supplementary S1: The formula of the radiomics signature

$$Radimocs\ score = -1 \times b + \sum_{i=1}^{N} (\boldsymbol{sv_i} \cdot \boldsymbol{x})$$

where

b being the intercept,

N being the number of support vectors,

$\boldsymbol{sv_i}$ being the ith support vector,

x being the new data, and X indicates the filtered radiomics features

$\cdot$ being the dot product.
